# Patients’ perceptions of oral cancer screening in dental practice: a cross-sectional study

**DOI:** 10.1186/1472-6831-12-55

**Published:** 2012-12-18

**Authors:** Oluwatunmise Awojobi, Suzanne E Scott, Tim Newton

**Affiliations:** 1 Unit of Social and Behavioural Sciences, King’s College London Dental Institute, Denmark Hill Campus, Caldecot Road, London, SE5 9RW, UK

**Keywords:** Oral Cancer, Early Detection of Cancer, Screening, Awareness, Public Health

## Abstract

**Background:**

Oral cancer is increasing in incidence in the UK and indeed worldwide. Delay in diagnosis is common; up to half of patients are diagnosed with advanced lesions. Thus it is essential to develop methods to aid early detection. This study aimed to assess dental patients’ experiences and awareness of oral cancer and screening within general dental practice.

**Methods:**

A cross-sectional questionnaire survey of 184 English-speaking adults, with no previous history of oral cancer was conducted. The questionnaire collected data on participant’s knowledge of oral cancer, experience of ‘screening’, attitudes and feelings towards having a screening, anticipated help-seeking behaviours, health-related behaviours (particularly risk factors) and sociodemographics.

**Results:**

Twenty percent of respondents had never heard of oral cancer; 77% knew little or nothing about it and 72% did not know that their Dentist routinely screens for oral cancer. Overall, attitudes to screening were positive. Ninety two percent of respondents would like their Dentist to tell them if they were being screened for signs of oral cancer and 97% would like help from their Dentists to reduce their risk.

**Conclusion:**

Patients seem generally unaware of oral cancer screening by their dentist but are happy to take part in screening, would like to be informed, and welcome the support of their Dentist to reduce their risk of developing oral cancer.

## Background

Oral cancer refers to cancers affecting the mouth, lip and oral cavity. The Tumour-Node-Metastasis (TNM) staging system for Head and Neck Cancers is employed to describe how advanced the cancer is, depending on the size of the tumour, whether regional lymph nodes have been affected or the cancer has spread to a different part of the body (metastasis). The stage at which oral cancer is diagnosed is a major determinant of mortality and morbidity following treatment [1]. For instance, Stage 1 (early disease) has an 80% 5-year survival rate whereas for Stage 4 (advanced) disease the 5 year survival rate can be as low as 20% [2]. The most recent statistics shows that worldwide there were 263, 900 new cases and 128,000 deaths in 2008 and the incidence of oral cancer is on the increase across the world in both developed and developing countries and regions including Melanesia, South-Central Asia and Eastern Europe [3]. In the United Kingdom, the latest yearly incidence of oral cancer had risen to 6,236 cases as of 2009 with a mortality rate of 1985 across the country in 2010 [4]. Detecting oral cancer at an early stage is the most effective means of improving survival and reducing morbidity from the disease [5].

The two major known risk factors for oral cancer are alcohol and tobacco. These factors have a synergistic effect so people who both drink and use tobacco have a much higher risk of oral cancer than those using only alcohol or tobacco [6]. Other factors that have been implicated in the development of oral cancer include poor diet and nutrition, sun exposure and the human papilloma virus [7]. Oral cancer is most common in males, lower socioeconomic groups and in ethnic minority groups [8] although rates in females are on the rise with an average increase of 3% each year since 1989 [4]. The majority of oral cancers are diagnosed in patients over 40 years of age [9].

Early diagnosis is ensured by the prompt response of patients and healthcare professionals to early signs and symptoms in order to facilitate diagnosis and treatment before the disease becomes advanced. However, approximately 30% of patients wait more than three months before consulting a healthcare professional about signs of oral cancer [10]. Delayed presentation has been found to be influenced by the process of symptom interpretation, knowledge of oral cancer, coping responses and barriers to seeking help such as problems with access and their social circumstances and responsibilities [11]. However, lack of awareness leading to misattribution of symptoms, has been reported as the most common reason for delay in seeking help for oral cancer signs and symptoms [12]. This lack of awareness about oral cancer in the UK has been reported by Warnakulasuriya [13] and awareness of the early signs of the disease was found to be low (except for persistent ulcers) [14]. Furthermore, awareness has been found to be lower in individuals at higher risk [15].

Early diagnosis of oral cancer could be aided by opportunistic screening for signs and symptoms among patients attending for routine dental care in primary care settings. In the UK, Speight et al. [16] and Johnson et al. [17] propose that targeted opportunistic oral cancer screening of high risk individuals attending a General Dental Practice may be the most cost-effective option for oral cancer screening. Such screening involves a simple systematic visual examination of the oral cavity and includes palpation of the head, neck and soft tissues [18]. During a dental check-up, it is routine practice for a soft tissue examination to be carried out for all patients, when the oral mucosa is inspected and oral tissues are palpated (preferably including lymph nodes). As this is the case for all patients, it is assumed that screening for oral cancer is also done routinely when high risk patients attend the dental practice.

Informing high risk patients that they are being checked for early signs of oral cancer during a routine examination could present a prime opportunity to provide this group with information about the existence of oral cancer and advice surrounding prevention and early detection (which includes interpretation of signs, symptoms and prompt help-seeking). The British Dental Association (BDA) does advise that patients should normally be told that an oral cancer check is being carried out [16]. However, patients are often unaware that they have been screened for signs of oral cancer [19]. Data from the U.S indicates that General Dental Practitioners (GDPs) are reluctant to tell their patients they are performing an oral mucosal examination and often avoid using the word ‘cancer’ altogether as they are concerned about alarming patients [20]. GDPs in the U.S. have also suggested that patients may not be receptive to information about oral cancer; however, patients appear to be in favour of discussing oral cancer with their Dentists [20].

In order to explore the extent of the missed opportunity for increasing oral cancer awareness in dental practices, this study surveyed a sample of adults attending two General Dental Practices in South East London. The aims were to explore patients’ awareness of oral cancer and oral cancer examination experiences including whether such screening or results of the screening were discussed with them. In addition, this study compared knowledge of the signs of oral cancer, anticipated help-seeking behaviours and oral cancer examination experiences between those who are at low and high risk of developing oral cancer.

## Method

### Participants

Participants were English speaking adults over 18 years of age, with no previous history of oral cancer. Participants were invited to take part from two General Dental Practices in two boroughs in South East London. The two boroughs were selected because each of the boroughs had an estimated adult smoking prevalence for 2006–08 that was higher (27%) than the regional average for London (21%) [21]. The Dental Practices were approached based on their practice location, size and on previous working relationships with the department conducting the research. A total of ten dentists work across both practices from which patients were recruited.

### Procedure

The questionnaire was piloted for face validity by staff and patients at the King’s College London Dental Institute, which has a patient population similar to the study sample. No major amendments were required following feedback from the pilot. Initial patient contact was made by sending an invitation letter from the principal dentist and a detailed information sheet to all patients who had appointments during the study period. The information sheet included the purpose of the study which was to know the current levels of patient knowledge and awareness as well as what was required of participants. The survey was self-administered, voluntary and anonymous. All patients had the option to either complete the questionnaire at the surgery and return it immediately or take the questionnaire home to complete at their own convenience. Participants who chose the latter option were given a freepost envelope with which to return the completed questionnaire. Reminder letters including new copies of the questionnaire and return envelopes were sent to all respondents whose completed questionnaires had not been received within four weeks. It was clearly stated in the information sheet that returning a completed questionnaire implied consent. All data was collected over a period of ten days between April and June 2011.

### Ethical considerations

Ethical approval for this study was received from London – Bloomsbury National Research Ethics Service Committee [22]. It is possible that if Dentists at participating practices were informed that patients will be asked if they are aware that the Dentist checked their mouth for any signs of oral cancer and if the results of this screening were discussed with them, the Dentists may have altered their own behaviour in consultations. The British Psychological Society has guidelines which allow for the withholding of some of the details of study if such knowledge of these details is likely to lead to a modification of behaviour [23] although debriefing should occur as soon as possible after data has been collected. Following these guidelines, Dentists in this study were informed that there was a survey of their patients’ knowledge and awareness about oral cancer but they were not made aware of the aspect of the questionnaire on oral cancer screening experience until after all data had been collected.

### Measures

The questionnaire was based on the validated measures developed and used in a similar study in the United States as well as those used in the United Kingdom. It was divided into five sections and collected data on key information about participant’s knowledge, experience and awareness about early detection of oral cancer along with health-related behaviours (particularly risk factors for oral cancer), and socio-demographic details, including age, gender, ethnicity, education, marital status and socio-economic status. An additional file shows the full questionnaire [see Additional file 1]. The entire questionnaire took about fifteen minutes to complete.

#### Healthcare use

The questionnaire also asked respondents asked about their use of healthcare services. There were questions enquiring about when they last visited their GP as well as how much time had passed between their previous visit to the Dentist and the current appointment. Their reasons for visiting the Dentist were also explored.

#### Health-related behaviours: Risk factors

##### Alcohol use

The 3-item Alcohol Use Disorders Identification Test Consumption (AUDIT-C) was used to assess alcohol use. This is a short-form version of the Alcohol Use Disorders Identification Test (AUDIT), a screening tool which was developed by the World Health Organization [24]. The AUDIT-C is used worldwide for identification of alcohol misuse and has been validated [25]. The questions asked include how often alcohol is consumed and the quantity consumed. Total Audit-C scores are calculated with a score of five or more indicating high risk.

##### Tobacco use

Using items from the questionnaire by the Office for National Statistics in conjunction with the Department of Health and the NHS Information Centre for Health and Social care [26], respondents were asked if they smoke at all nowadays or if they did so in the past. There were also questions regarding quantity of cigarettes smoked and other tobacco use like chewing tobacco.

#### Knowledge and experience of oral cancer and screening

Participants’ self-reported knowledge and awareness of oral cancer was elicited by asking if they had heard of the disease and how much they knew about it, ranging from a lot to nothing at all [19]. Participants’ awareness was probed further by asking whether or not they knew if their mouths had ever been screened for oral cancer, if this was done by their Dentist and when. There was also one question regarding their awareness of any extra oral examination of lymph nodes. There were three possible answers to these questions, ‘Yes’, ‘No’ and ‘Don’t Know/Not sure’. For some of the analysis, respondents answering ‘No’ or ‘Don’t Know/Not Sure’ were grouped together as unaware while those answering ‘Yes’ were aware. Additionally, two subscales from the Humphris Oral Cancer Knowledge Scale [15] explored respondents’ knowledge of risk factors for oral cancer as well as their knowledge of what screening for oral cancer entails.

#### Attitudes and emotion towards screening for oral cancer

##### Attitudes towards oral cancer ‘screening’

Patients’ attitude towards having an oral cancer screening was also investigated using four items from the Humphris Oral Cancer Knowledge Scale. A ‘total’ score for attitude was derived by summing the individual scores for each question. The lowest possible score (0) means a very negative attitude to screening and the highest possible score is 16 reflecting a very positive attitude to screening.

##### Emotion towards oral cancer ‘screening’

A subscale describing respondents’ feeling towards having a check up for mouth cancer was included. Participants were asked to rate how anxious, concerned and worried they would be on a likert scale ranging from ‘not anxious’ to ‘extremely anxious’. Scores range from 0 (low emotion) to the highest score of 12 (high emotion).

#### Patients’ desire to know if screening is taking place and need for support to reduce risk

Two questions were asked specifically to determine patients’ desire for information and communication about oral cancer screening and risk management. The first question was ‘Would you want your dentist to *tell* you if they were *checking* your mouth for signs of mouth cancer?’ and the second was ‘Would you want your dentist to *help you reduce your risk* of getting mouth cancer’. Responses were ‘Yes’, ‘No’ and ‘Don’t know/Not sure’.

#### Anticipated help-seeking behaviour

In order to ascertain respondents’ intentions to seek help for possible signs of oral cancer, they were asked if they would seek help for a list of twelve signs assuming these signs had persisted for three weeks or more. These signs included five signs (a red patch, a white patch, a painful ulcer, swelling in the mouth and pain in the mouth) commonly associated with oral cancer. They were also asked to choose which health care professional they would go to for help concerning these signs should it persist for more than three weeks. The questions used in this section are a modification of a questionnaire that was developed by Scott et al. [27]. The term ‘Anticipated delay’ is used to refer to a situation wherein respondents do not intend to visit a Doctor or Dentist for signs associated with oral cancer that had lasted for three weeks.

### Analysis

Descriptive statistics were used to describe the sample, their knowledge and experiences. Inferential statistics were then used to check for relationships between outcome measures and risk factors (e.g. alcohol use and smoking status), sociodemographics (borough, gender, age, marital status, ethnicity, educational qualification and socioeconomic classification) as well as health behaviours (e.g. visiting the GP, visiting the Dentist and the reason for Dental visit).

Sample size was calculated by conducting power analysis using the statistical software G Power version 3.0.5. The sample size was based on providing sufficient power for t-tests, Chi-square tests for 1 degree of freedom and Pearson correlation analysis. To compensate for missing data, recruitment continued until 186 participants returned their questionnaires. The software used for analyzing the data was the Statistical Package for Social Sciences version 19.

## Results

### Response rate

Out of a possible 362 eligible adults who were approached to take part in the survey, 186 adults (51%) completed and returned their questionnaires. Two respondents were excluded from the final analysis because they reported having had oral cancer previously. Table 1 has a summary of the socio-demographic characteristics of participants.

**Table 1:** Socio-Demographic Characteristics of Participants

	**Frequency**	**Percent**
**Age**		
18 to 39 years old	59	36.9
40 year old and over	101	63.1
**Gender**		
Male	67	37.4
Female	112	62.6
**Ethnicity**		
White	85	47.8
Black	63	35.4
Other	30	16.9
**Marital Status**		
Single, never married	75	41.7
Married and living with spouse	53	29.4
Married but separated from spouse	14	7.8
Divorced	22	12.2
Widowed	8	4.4
Other*	8	4.4
**Educational Qualification**		
Degree or Degree Equivalent	63	41.7
Higher Educational Qualification (below degree)	12	7.9
A Levels, Vocational Level 3 & Equivalents	30	19.9
Trade Apprenticeships	27	17.9
Qualifications at Level 1 or below	4	2.6
Other Qualification: Level Unknown	3	2.0
No Qualifications	12	7.9
**National Statistics Socio-economic Classification**		
Managerial and professional occupations (Level 1)	89	48.4
Intermediate occupations (Level 2)	16	8.7
Small employers and own account workers (Level 3)	10	5.4
Lower supervisory and technical occupations (Level 4)	14	7.6
Semi-routine and routine occupations (Level 5)	19	10.3
Students, occupations not stated or not classifiable	36	19.6

*3 “Co-habiting”, 1 “Civil Partnership”, 2 “Partner” (No further details provided), 2 Non responses.

Chi square tests indicated that there were no significant variations between participants recruited from the two boroughs apart from respondents from the Southwark practice being more likely to be under 40 years of age (69.5%) compared to those recruited from the Lewisham practice (30.5%) (*X*^2^ = 7.281, p= 0.007).

### Healthcare utilization

Seventy-eight percent of participants reported visiting their Dentist within the past year in addition to their current visit. A higher proportion, (89%), reported visiting their GP within the same period. Of all the respondents, 50% reported visiting their Dentists for regular checkups, 18% for occasional checkups and 34% only visit the Dentist when having trouble with their teeth.

### Health-related behaviours: risk factors

#### Alcohol use

Twenty three percent of respondents reported never having a drink that contains alcohol while 30% reported having alcoholic drinks two to three times a week or more.

Alcohol Use Disorders Identification Test Consumption (Audit-C) scores were calculated for 118 respondents who had sufficient data to compute the score. The mean Audit-C score was 3.93 (95%CI 3.53, 4.33) with a median of 4 and range 1 to 10. Thirty seven percent (N=44) of respondents had a score of 5 or more which is the high risk category.

#### Tobacco use

Table 2 shows current and lifetime tobacco use as well as quantities smoked. The proportion of ever-smokers in this sample was 58%, with 24% of respondents reporting being current smokers and 34% being previous smokers who had quit. Of those who had quit, 35% stopped smoking more than 10 years ago. About 10% of ever-smokers (current or ex-smokers) reported having their first cigarette less than five minutes after waking up but the majority (53%) had their first cigarette about an hour or more after waking. Four respondents (2%) reported chewing some form of tobacco. On average respondents smoked more during the weekends than on weekdays with a mean value of 13.44 cigarettes smoked during the weekends compared to 10.49 during the week.

**Table 2:** Smoking Status and Cigarette Quantity per Day (Cigarette Sticks)

		**Frequency**	**Percent**
**Current Smoking Status**			
**Yes, I smoke cigarettes**		43	23.9
**No, but I used to smoke cigarettes**		62	34.4
**No I have never smoked cigarettes**		75	41.7
**Cigarette Quantity per Day (Sticks)**			
	**Weekdays**	**Weekends**	**Combined**
**Mean**	10.49	13.44	11.33
**Median**	10	10	9.29
**Std. Deviation**	7.72	10.78	8.09
**95% CI**	8.84, 12.16	11.08, 15.74	9.60, 13.07
**Range**	0 - 30	0 - 60	1 - 33

### Knowledge and awareness of oral cancer

Seventy three percent of respondents reported having heard of oral cancer while 20% said they had not and 7% were unsure if they had heard of it or not. When asked how much they knew about the disease, only 6% reported that they knew ‘a lot’, and the vast majority (77%) reported that they knew ‘a little’ or ‘nothing at all’ (Table 3).

**Table 3:** Self-reported Knowledge of Oral Cancer

	**Frequency**	**Percent**
***Have you ever heard of mouth cancer?***		
Yes	**134**	**73**
No	**36**	**20**
Dont know/Not sure	**13**	**7**
***Would you say you know a lot, some, a little, or nothing at all about mouth cancer?***		
A lot	5	3
Some	29	16
A little	65	36
Nothing at all	76	41
Never heard of mouth cancer	8	4

The mean oral cancer knowledge score (risk factor subscale) was 9.96 (95% CI 9.79, 10.28) with a range of 3 to 12 items answered correctly out of 13 items. The mean oral cancer knowledge score (screening subscale) was 6.09 (95% CI 5.95, 6.31) with a range of 2 to 7 items answered correctly out of 7 items.

### Emotion and attitudes towards screening for oral cancer

Thirty nine percent of respondents said they would not feel anxious, worried or concerned about having their mouths checked for signs of oral cancer with a further 25% indicating they would only feel a little anxious, worried or concerned. The mean emotion score was 2.52 (95%CI 2.01, 2.88). Only a minority (1%) reported extreme levels of anxiety, worry and concern about oral cancer screening. With regards to attitudes, overall there was a generally positive attitude to screening with a mean score of 13.04 (95%CI 12.68, 13.41). Approximately 21% of respondents had very positive attitudes to being screened obtaining the highest possible score of 16.

### Experience of oral cancer screening

Table 4 summarizes the data regarding participants’ awareness of oral cancer screening. When asked whether Dentists were trained to check for signs of oral cancer more than half (56%) of respondents said they did not know or were unsure. Furthermore, 72% also did not know if their own Dentist screens them for signs of oral cancer as part of their routine check up, and 60% said they did not know if their mouths had ever been checked for signs of oral cancer by any Dentist. Consequently, when asked when their most recent check took place 56.4% reported not knowing and 27.1% said that their mouths had never been checked. Only 13% indicated that their chin or neck had ever been felt as part of their examination. Of those who recall this extra oral examination, 44% said they received an explanation for this.

**Table 4:** Awareness of Oral Cancer Examination

	**Frequency**	**Percent**
**Aware that Dentists are trained to check for signs of oral cancer**		
Yes	75	43
No	4	2
Don’t Know/Not Sure	102	56
**Awareness of screening routinely by *their *Dentist**		
Yes	26	14
No	25	14
Don’t Know/Not Sure	131	72
**Awareness of *ever *being screened by *any *Dentist**		
Yes	21	12
No	52	28
Don’t Know/Not Sure	110	60
**Most recent screening for signs of oral cancer**		
Today (Current Visit)	15	8
Within the past year	9	5
1 to 2 years ago	2	1
2 to 3 years ago	1	1
Over 3 years ago	3	2
Don’t know/Not Sure	102	56
My mouth has never been checked	49	27
**Awareness of extra oral examination**		
Yes	24	13
No	129	71
Don’t Know/Not Sure	30	16
**Received an explanation for extra oral examination**		
Yes	10	44
No	9	39
Don’t Know/Not Sure	4	17
**Patients want to be told Oral Cancer Screening is taking place**		
Yes	163	92
No	9	5
Don’t Know/Not Sure	6	3
**Patients want support to reduce their risk of Oral Cancer**		
Yes	176	97
No	2	1
Don’t Know/Not Sure	3	2

Ninety-two percent of respondents indicated that they would like their Dentists to tell them if their mouths were being checked for signs of oral cancer. Only 5% did not want to be told and 3.4% were unsure. Moreover, 97% said they would like help from their Dentists to help them reduce their risk of getting oral cancer.

### Help-seeking behaviour

Proportions of participants reporting that they would visit their Doctor rather than their Dentist for individual signs of oral cancer were 62% for white patch, 61% for red patch, 51% for an ulcer and 54% for a swelling. This trend was reversed in the case of pain in the mouth where the majority (50.9%) indicated they would visit the Dentist. Seventy-seven percent of participants anticipated delaying seeking help for at least one sign of oral cancer.

### Relationships between variables, sociodemographics and health behaviours

There was a small but significant correlation between alcohol use and attitudes to screening (rho=0.154, p=0.047) as well as knowledge about oral cancer screening (rho=0.226, p=0.004). Those who drank alcohol had a more positive attitude to screening and had more accurate knowledge about what screening for oral cancer entails. Similarly, ever smokers were found to have more positive attitudes to being screened for oral cancer (rho=0.157, p=0.042) compared to those who had never smoked. However, ever smokers were less aware that their Dentist screened them routinely for signs of oral cancer (rho= −0.151, p=0.044). Older participants (those 40 years and older) were less worried about being checked for signs of oral cancer by their Dentist than those in the younger age group (rho= −0.167, p=0.049). Married participants were more likely to want to know if their Dentist was screening them for signs than non- married (single, divorced or widowed) (rho= 0.218, p=0.004).

A Mann–Whitney *U* test revealed white respondents were less emotional about screening (Md = 0) than black respondents (Md = 3) as well as respondents from other ethnic backgrounds (Md = 2) (z= 13.250, p = 0.001). White people were also significantly more knowledgeable about what screening entails (z = 12.882, p = 0.002) than the other ethnic groups, with a median value of 7 compared to a slightly lower score of 6 for Black and Other ethnic groups. Additionally patients who had previously heard of oral cancer had more positive attitudes to screening (rho=0.189, p=0.014). A Kruskal-Wallis test revealed statistically significant differences in attitudes to screening as well as knowledge of screening between participants with different socioeconomic classification. Those in intermediate occupations had more positive attitudes (Md = 14.50) compared to managerial and professional occupations (Md = 12) or semi-routine/routine occupations (Md = 13) (*X*^2^ = 16.271, p=0.006). Similarly, respondents who were classified as having intermediate occupations also had the highest score for knowledge of what an oral cancer screening entails with a median score of 7 whereas all other groups had a median score of 6 (*X*^2^=11.417, p=0.44). Finally, a Mann–Whitney *U* test revealed respondents who went for regular checkups had significantly more positive attitudes to screening(Md= 13.50) than those who went occasionally (Md=12.36) or only with trouble (Md=12.97).

## Discussion

This exploratory study sought to document the extent of the missed opportunity for increasing oral cancer awareness within dental practices. In similarity to the findings in the United States [19] a low proportion of participants in this study were aware that they had been checked for oral cancer by their dentist (at their last appointment or ever). In, addition those patients with risk factors (age, tobacco use, alcohol consumption) were not any more aware that oral cancer screening is conducted by their dentist, compared to those without such risk factors.

Respondents who were more at risk (via alcohol use, smoking status) were also significantly more likely to have positive attitudes to screening. Furthermore, higher risk patients are said to visit the Dentist less regularly than low risk patients [28], however the results of the current study indicate that regularity of visit does not affect attitudes to screening. There was no significant difference in attitudes between those who visited their Dentist within a year and those that visited more than two years ago. Furthermore, unlike the study by West et al. [14] that found only 4.4% reported not having heard of oral cancer, in this study 19.7% said they had not heard of oral cancer. This gives cause for concern as low levels of awareness will affect the chances of early presentation.

Using the opportunity provided by a dental appointment to raise awareness may be increasingly vital to encouraging early detection of oral cancer. This sample of patients reported very positive attitudes towards screening for oral cancer (including being told that this screening is taking place) and low levels of negative emotion about being screened. This is a positive message from participants and this supports data from the United States. Choi et al. [20] also found patients would like their Dentists to tell them if they were being screened for signs of oral cancer. A contradiction however can be seen in that same study as Dentists expressed concern that screening will make patients anxious.

Symptom misinterpretation is a common reason for delayed diagnosis [11]. Overall, a high percentage of patients anticipated a delay in seeking help for at least one sign of oral cancer in this survey with patients intending to seek help for an average of 2.4 of the five signs presented. This could contribute considerably to a delay in diagnosis and treatment. This study also found that a higher proportion of participants reported that they intended to seek help from a Doctor than from a Dentist for the signs of oral cancer. There is a need to ensure Doctors are specifically trained in referral and care pathway for oral cancers so that they are equipped to adequately support their patients and not contribute further to any delay.

An additional finding is that there are differences in knowledge, awareness and experiences between groups according to their sociodemographics (e.g. ethnicity) and other variables. For instance, white people were more knowledgeable about what screening entails and ever smokers were less aware that their Dentists screened them routinely. This highlights the fact that interventions and strategies may need to be targeted more closely to certain groups.

This study has some limitations that should be considered. One limitation is the response rate (51%). It is difficult to demonstrate that the people who chose not to participate in the study were not significantly different from the people who did. Findings need therefore to be interpreted with caution. However many of those recruited had risk factors for oral cancer and had poor knowledge of oral cancer. This sample’s risk status does not appear to be much different from that of the wider local population (21). Current smoking rates in this study (23.9%) are higher than that for London (20.8%) but slightly lower than most recent rates reported for Lewisham (27.1%) and Southwark (26.9%) [21]. However, 60% of all respondents had ever smoked, which means a high proportion are at an increased risk of getting oral cancer when other factors like age (63% over 40 years of age) and alcohol use (77% consumed alcohol) are taken into account.

Additionally, this study did not set out to determine whether dentists are performing full and thorough oral cancer screening with high risk patients, however it does indicate that patients are unaware that oral cancer screening is taking place. Kujan et al. [29] reported over 95% of GDPs and Specialist Dentists reported using a visual examination for oral cancer screening. In a previous study of 2519 UK Dentists [30], 84% reported that they perform screening of the oral mucosa routinely in practice and this figure was 82% in a more recent US Study [31]. Based on these figures it is likely screening may indeed be taking place but the gap is found in that Dentists may not be communicating this fact to their patients.

## Conclusion

This study has indicated that patients seem to be generally unaware of oral cancer screening by their dentist yet they are happy to be informed that they are being screened and would like the support of their Dentist to reduce their risk of developing oral cancer. Patients did not express anxiety, concern or worry about receiving this information. Further research is required to explore the views of UK-based Dentists on how best to communicate with patients’ without increasing anxiety or prolonging dental consultations.

## Competing interests

The authors declare that they have no competing interests.

## Authors’ contributions

OA participated in the study conception and design as well as coordinated the study including data collection. OA also performed the statistical analysis and drafted the manuscript. SS and TN made substantial contributions to conception and design of the study and overall supervision of the project. In addition SS and TN also contributed to drafting of manuscript. All authors read and approved the final manuscript.

## Pre-publication history

The pre-publication history for this paper can be accessed here:

http://www.biomedcentral.com/1472-6831/12/55/prepub

## Supplementary Material

Additional file 1 **Mouth cancer awareness questionnaire.** This is a copy of the questionnaire that was used to collect data for this study. Click here for file
