# Supplementary material for: Patients’ perceptions of oral cancer screening in dental practice: a cross-sectional study
Source: BMC Oral Health. 2012 Dec 18;12:55. doi: 10.1186/1472-6831-12-55 (PMC3540027; doi:10.1186/1472-6831-12-55)
Supplement: Additional file 1 — Mouth cancer awareness questionnaire. This is a copy of the questionnaire that was used to collect data for this study. [file 1472-6831-12-55-S1.pdf]

Mouth Cancer Awareness in Dental Patients

- **Try to answer all the questions.**
- **All the information you give will be treated in the strictest confidence.**
- **Once you have completed the questionnaire you should return it to the researcher or post it using the stamped envelope provided.**
- **For further information please contact:**

'Mouth Cancer Awareness Study'
Attn: Tunmise Awojobi
Oral Health Services Research Unit
King's College London Dental Institute
Caldecot Road
Denmark Hill
London SE5 9RW
Tel 020 3299 4756

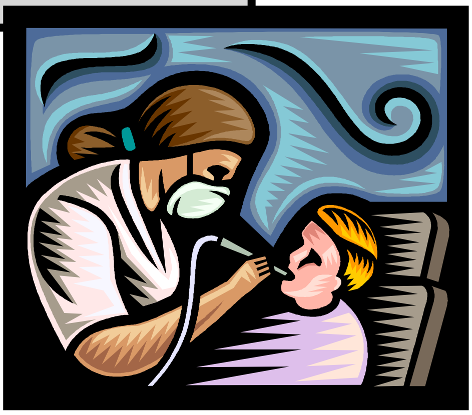

SECTION A: These questions are about your use of healthcare services

1. Before today's visit, how long ago was your last visit to the Dentist?

Within the past year	1 to 2 years ago	More than 2 years ago
❑	❑	❑

2. In general do you go to the Dentist for:

A regular check up	An occasional check up	Only when having trouble with your teeth
❑	❑	❑

3. When did you last visit your GP?

Within the past year	1 to 2 years ago	More than 2 years ago	Never been to the GP
❑	❑	❑	❑

4. For each of the following symptoms, please indicate which (if any) healthcare professional you would visit about that symptom. If you would not seek help for that symptom please tick the last column.

	Doctor (GP)	Dentist	Pharmacist	Other	I would not seek help for this symptom
**a) A white patch in the mouth that has lasted more than three weeks**	❑	❑	❑	❑	❑
**b) Dizziness that has lasted more than three weeks**	❑	❑	❑	❑	❑
**c) A red patch in the mouth that has lasted more than three weeks**	❑	❑	❑	❑	❑
**d) Stomach ache that has lasted more than three weeks**	❑	❑	❑	❑	❑
**e) A painful ulcer in the mouth that has lasted more than three weeks**	❑	❑	❑	❑	❑
**f) A yellow patch in the mouth that has lasted more than three weeks**	❑	❑	❑	❑	❑
**g) A rash on the face that has lasted more than three weeks**	❑	❑	❑	❑	❑
**h) A swelling in the mouth that has lasted more than three weeks**	❑	❑	❑	❑	❑
**i) A sore throat that has lasted more than three weeks**	❑	❑	❑	❑	❑
**j) A headache that has lasted more than three weeks**	❑	❑	❑	❑	❑
**k) Pain or discomfort in the mouth that has lasted more than three weeks**	❑	❑	❑	❑	❑

SECTION B: These questions are mainly about mouth cancer awareness

1. Have you ever heard of mouth cancer?

Yes	No	Don’t know/Not sure
❑	❑	❑

2. Would you say you know a lot, some, a little, or nothing at all about mouth cancer?

A lot	Some	A little	Nothing at all	Never heard of mouth cancer
❑	❑	❑	❑	❑

3. Are Dentists trained to check the mouth for signs of mouth cancer?

Yes	No	Don’t know/Not sure
❑	❑	❑

4. Does *your* Dentist check your mouth for signs of mouth cancer during routine dental appointments?

Yes	No	Don’t know/Not sure
❑	❑	❑

5. Has your mouth *ever* been checked for signs of mouth cancer by a Dentist?

Yes	No	Don’t know/Not sure
❑	❑	❑

6. When was your mouth last checked for signs of mouth cancer?

Today (This visit to the Dentist)	Within the past year	1 to 2 years ago	2 to 3 years ago	Over 3 years ago	Don’t know/ Not sure	My mouth has never been checked for signs of mouth cancer
❑	❑	❑	❑	❑	❑	❑

7. During your dental appointment, has your Dentist ever felt under your chin and around your neck?

Yes	No	Don’t know/Not sure
❑	❑	❑
(go to question 8)	*(go to question 9)*	*(go to question 9)*

8. Did they explain to you why they were doing this?

Yes	No	Don't know/Not sure
❑	❑	❑

9. Would you want your dentist to tell you if they were checking your mouth for signs of mouth cancer?

Yes	No	Don't know/Not sure
❑	❑	❑

10. Would you want your dentist to help you to reduce your risk of getting mouth cancer?

Yes	No	Don't know/Not sure
❑	❑	❑

11. Please indicate the extent to which you agree with each of the following statements:

a) The dentist checking my mouth for cancer will reassure me that everything is alright.

Strongly agree	Agree	Neither	Disagree	Strongly disagree
❑	❑	❑	❑	❑

b) The dentist checking my mouth for mouth cancer will be a waste of time.

Strongly agree	Agree	Neither	Disagree	Strongly disagree
❑	❑	❑	❑	❑

c) The dentist checking my mouth for mouth cancer will give me discomfort.

Strongly agree	Agree	Neither	Disagree	Strongly disagree
❑	❑	❑	❑	❑

d) The dentist checking my mouth will lead to early diagnosis of mouth cancer if I have it

Strongly agree	Agree	Neither	Disagree	Strongly disagree
❑	❑	❑	❑	❑

12. How do you feel about having a check for mouth cancer? (Answer the three ratings: "anxiety", "worry" and "concern" below)

**a)**	Not Anxious	Slightly anxious	Fairly anxious	Very anxious	Extremely anxious
	❑	❑	❑	❑	❑
**b)**	Not Worried	Slightly worried	Fairly worried	Very worried	Extremely worried
	❑	❑	❑	❑	❑
**c)**	Not concerned	Slightly concerned	Fairly concerned	Very concerned	Extremely concerned
	❑	❑	❑	❑	❑

13. For each of the following statements, indicate whether you think it is true or false.
If you are unsure, have a guess.

People are more likely to get mouth cancer if they:	True	False
a) Are over 50 years old	❑	❑
b) Smoke tobacco (cigarettes, cigars or pipe)	❑	❑
c) Drink strong tea	❑	❑
d) Drink strong 'filter' coffee	❑	❑
e) Drink decaffeinated coffee	❑	❑
f) Chew tobacco	❑	❑
g) Drink alcohol heavily	❑	❑
h) Wear dentures	❑	❑
i) Eat hazel nuts	❑	❑
j) Overeat	❑	❑
k) Are a man	❑	❑
l) Are a woman	❑	❑
m) Have lost all their teeth	❑	❑

14. A check up for mouth cancer by a Dentist:	True	False
a) Is painless	❑	❑
b) Is not required if you wear false teeth	❑	❑
c) Is carried out using x-rays	❑	❑
d) Is a way of finding mouth cancer at an early stage	❑	❑
e) Only takes a few minutes	❑	❑
f) Is carried out during routine dental check-ups	❑	❑
g) Is only necessary for people over 70 years old	❑	❑

SECTION C: The following questions are about your lifestyle.

1. How often do you have a drink that contains alcohol?

Never	Monthly or less	2 to 4 times a month	2 to 3 times a week	4 or more times a week
❑	❑	❑	❑	❑

2. How many standard alcoholic drinks do you have on a typical day when you are drinking?

0	1 to 2	3 to 4	5 to 6	7 to 9	10 or more
❑	❑	❑	❑	❑	❑

3. How often do you have 6 or more standard drinks on one occasion?

Never	Less than monthly	Monthly	Weekly
❑	❑	❑	❑

4. Do you smoke cigarettes at all nowadays?

Yes	No, but I used to smoke	No, I have never smoked
❑	❑	❑
(go to question 5)	(*go to question 5*)	(*go to question 9*)

5. How soon after waking do (or did) you smoke your first cigarette of the day?

Less than 5 minutes	5 to 14 minutes	15 to 29 minutes	30 minutes but less than 1 hour	1 hour but less than 2 hours	2 hours or more
❑	❑	❑	❑	❑	❑

6. How many cigarettes a day do (or did) you usually smoke at weekends?

____

7. How many cigarettes a day do (or did) you usually smoke on weekdays?

____

8. If you have stopped smoking cigarettes regularly, how long ago did you stop?

6 months to a year ago	1 to 2 years ago	2 to 5 years ago	5 to 10 years ago	More than 10 years ago
❑	❑	❑	❑	❑

9. Do you use any other type of tobacco (e.g. chewing tobacco)?

Yes	No
❑	❑

SECTION D: About You

1. What is your gender?

Male	Female
❑	❑

2. What was your age on your last birthday?

____

3. What is your marital status?

Single, that is, never married	Married and living with your husband/wife	Married and separated from your husband/wife	Divorced	Widowed	Other (Please Specify)
❑	❑	❑	❑	❑	____________

4. To which of these ethnic groups do you belong?

White	Mixed	Asian or Asian British	Black or Black British	Chinese	Other ethnic group
❑	❑	❑	❑	❑	❑

5. What is your highest educational qualification?

____

6. Have you ever had mouth cancer?

Yes	No
❑	❑

SECTION E: The following questions are about your job

1. **Have you *ever* worked?**

Yes ❑ (go to question 2)

No ❑ (go to PAGE 10)

The questions below refer to your current main job, or (if you are not working now) to your last main job. Please tick <u>one box only</u> for each question.

2. **What is (was) your main occupation?**

____

3. **Do (or did) you work as an employee or are (were) you self-employed?**

Employee ❑

Self-employed with employees ❑

Self-employed / freelance without employees ❑ *(go to question 6)* ➡

4. ***For employees*: indicate below how many people work (worked) for your employer at the place where you work (worked).**

***For self-employed*: indicate below how many people you employ (employed). Go to question 4 when you have completed this question.**

1 to 24 ❑

25 or more ❑

5. **Do (or did) you supervise any other employees?**

A supervisor or foreman is responsible for overseeing the work of other employees on a day-to- day basis

Yes ❑

No ❑

6. **Please tick one box to show which best describes the sort of work you do.**

(If you are not working now, please tick a box to show what you did in your last job).

PLEASE TICK ***<u>ONE BOX ONLY</u>***

❑ **Modern professional occupations**

(*such as:* teacher - nurse - physiotherapist - social worker - welfare officer - artist - musician - police officer (sergeant or above) - software designer)

❑ **Clerical and intermediate occupations**

(such as: secretary - personal assistant - clerical worker - office clerk - call centre agent - nursing auxiliary - nursery nurse)

❑ **Senior managers or administrators**

(usually responsible for planning, organising and co-ordinating work and for finance. Such as: finance manager - chief executive)

❑ **Technical and craft occupations**

(such as: motor mechanic - fitter - inspector - plumber - printer -tool maker - electrician - gardener - train driver)

❑ **Semi-routine manual and service occupations**

(*such as: postal worker - machine operative - security guard - caretaker - farm worker - catering assistant - receptionist - sales assistant)*

❑ **Routine manual and service occupations**

(such as: HGV driver - van driver - cleaner - porter - packer - sewing machinist - messenger - labourer - waiter / waitress - bar staff)

❑ **Middle or junior managers**

(such as: office manager - retail manager - bank manager - restaurant manager - warehouse manager – publican)

❑ **Traditional professional occupations**

(such as: accountant - solicitor - medical practitioner - scientist -civil / mechanical engineer)

Read the statement below and *tick the box* provided to give consent

Data Protection Statement

I consent to the processing of my personal information for the purposes explained to me. I understand that such information will be treated in accordance with the terms of the Data Protection Act 1998

❑

THANK YOU FOR YOUR TIME

Return the completed questionnaire to the researcher

or post it to us using the stamped envelope provided.
